# Structural proof of a [C–F–C]^+^ fluoronium cation

**DOI:** 10.1038/s41467-021-25592-6

**Published:** 2021-09-06

**Authors:** Kurt F. Hoffmann, Anja Wiesner, Carsten Müller, Simon Steinhauer, Helmut Beckers, Muhammad Kazim, Cody Ross Pitts, Thomas Lectka, Sebastian Riedel

**Affiliations:** 1 Fachbereich Biologie, Chemie, Pharmazie, Institut für Chemie und Biochemie – Anorganische Chemie, Freie Universität Berlin, Berlin, Germany; 2 Department of Chemistry, Johns Hopkins University, Baltimore, MD USA; 3 Present Address: Department of Chemistry, University of California, Davis, Davis, CA USA

**Keywords:** Chemical bonding, Structure elucidation

## Abstract

Organic fluoronium ions can be described as positively charged molecules in which the most electronegative and least polarizable element fluorine engages in two partially covalent bonding interactions to two carbon centers. While recent solvolysis experiments and NMR spectroscopic studies on a metastable [C–F–C]^+^ fluoronium ion strongly support the divalent fluoronium structure over the alternative rapidly equilibrating classical carbocation, the model system has, to date, eluded crystallographic analysis to confirm this phenomenon in the solid state. Herein, we report the single crystal structure of a symmetrical [C–F–C]^+^ fluoronium cation. Besides its synthesis and crystallographic characterization as the [Sb_2_F_11_]^−^ salt, vibrational spectra are discussed and a detailed analysis concerning the nature of the bonding situation in this fluoronium ion and its heavier halonium homologues is performed, which provides detailed insights on this molecular structure.

## Abstract (web summary)

Unlike other halogen atoms, the ability for fluorine to exist in a [C–X–C]^+^ connectivity pattern has only been shown in spectroscopic studies. Here the authors present a single crystal structure of a fluoronium cation, characterized by X-ray diffraction.

## Introduction

**Fig. 1:**
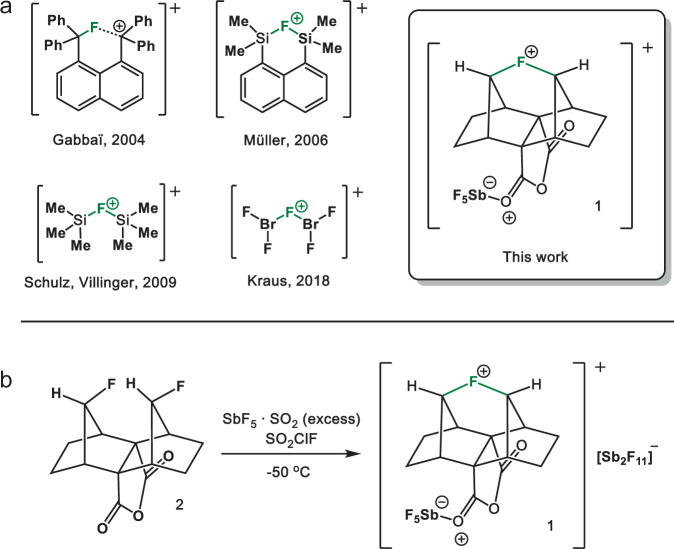
Overview of fluoronium ions in the condensed phase. **a** Crystallographically characterized fluoronium ions^16,17,19–23^. Note that the formal charges shown inside a circle do not represent the actual charge of the corresponding atoms. **b** Synthesis of the fluoronium salt [**1**][Sb_2_F_11_].

According to IUPAC, halonium ions are defined as ions of the form [R_2_X]^+^, where X may be any halogen^1^. In the case of organic halonium ions, R is defined as a cyclic or open-chained hydrocarbon backbone. Since they were first discussed as reactive intermediates in organic halogenation reactions in 1937^2^, a large variety of stable and structurally characterizable iodo-^3,4^, bromo-^5,6^, and chloronium^7–9^ salts of the type [C–X–C]^+^ have been synthesized^10,11^. On the other hand, fluoronium cations, in which a divalent fluorine atom (as depicted in a simplifying Lewis dot structure) is symmetrically bound to two carbon atoms, have only been reported thus far in spectroscopic investigations. For instance, Morton et al. first detected a three-membered cyclic fluoriranium ion as an intermediate in mass-spectrometry experiments^12^, while Gabbaï and coworkers obtained the structure of a diphenylnaphthylmethylium cation that shows an intramolecular bonding interaction to an adjacent fluorine substituent, allowing a description as an unsymmetrically bridged fluoronium cation (Fig. 1a)^13^.

In 2013, Lectka et al. presented the transient generation of a symmetrically bridged fluoronium cation in solution starting from a rigid double-norbornyl type precursor. Its formation as a fleeting reactive intermediate was indicated through isotopic labeling experiments^14,15^. Finally, in 2018 they supported the formation of the aforementioned fluoronium ion by NMR spectroscopy^16,17^; yet, the structural proof of this organic fluoronium ion in the solid state remained a lofty goal. In addition to these few spectroscopic examples of carbon-based fluoronium cations, some inorganic fluoronium cations have been investigated in the past. Motz and Bartmann published in 1988 a crystal structure of the simplest fluoronium ion [H_2_F]^+^ ^18^. A crystal structure of a cyclic disilylfluoronium salt was reported by Müller and coworkers in 2006, followed by the structure of an open-chained bissilylated fluoronium cation by Schulz in 2009^19–21^. More recently in 2018, Kraus presented examples of a fluorine atom coordinated by two BrF_2_ units (Fig. 1a)^22,23^.

In this work, we present a modified synthesis and structural investigation of the carbon-based double-norbornyl type fluoronium ion **1** (Fig. 1) as the [Sb_2_F_11_]^−^ salt by single-crystal X-ray diffraction. Furthermore, the bonding schemes of [C–X–C]^+^ (X = F, Cl, Br, I) are discussed and compared through detailed AIM analyses, and the properties of **1** are further analyzed by vibrational spectroscopy.

## Results

### Synthesis and characterization

Our approach is, in principle, based on utilizing the strong Lewis acid SbF_5_ as a fluoride ion abstractor^16,17^. Herein, neat SbF_5_ was substituted by the crystalline solvent-adduct SbF_5_⋅SO_2_ due to its slightly weakened acidic character and more convenient handling (Fig. 1b). By adding precursor **2** to a cooled mixture of SbF_5_⋅SO_2_ in SO_2_ClF, a yellow solution is formed. Partial evaporation of the SO_2_ClF and consecutive slow cooling of the reaction mixture afforded single crystals suitable for X-ray diffraction.

**Fig. 2:**
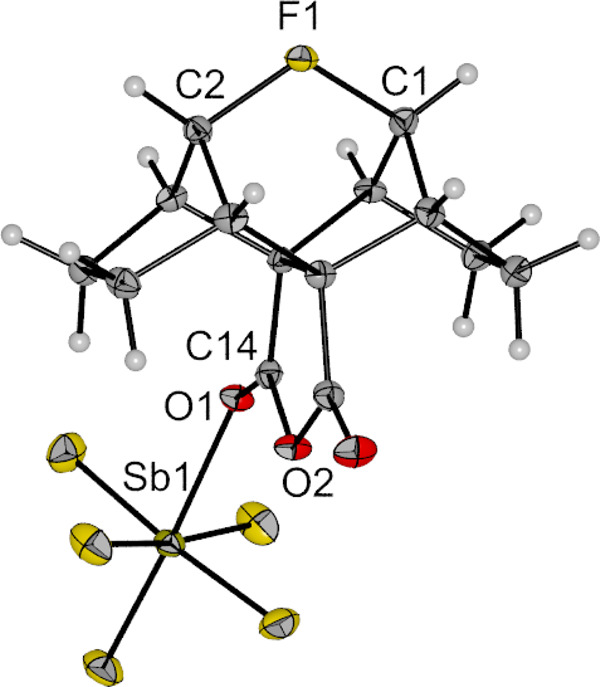
Molecular structure of the fluoronium ion 1 as its [Sb_2_F_11_]⋅(SO_2_ClF)_3_ salt in the solid state. Anion and solvent molecules are not depicted. Thermal ellipsoids set to 50% probability. Selected bond lengths [pm] and angles [°]: F1–C1 156.6(3), F1–C2 158.7(3), C1–F1–C2 115.64(17), O2–C14–O1–Sb1 19.0(4).

The compound [**1**][Sb_2_F_11_]⋅(SO_2_ClF)_3_ (Fig. 2, more detailed structure in Supplementary Fig. 1 including a comprehensive list of crystal data in Supplementary Tables 1–3) crystallizes in the centrosymmetric monoclinic space group *P*2_1_/*c* along with three solvent molecules per asymmetric unit. A nearly symmetrical C–F–C bonding array is observed. The bridging fluorine atom F1 and its adjacent carbon atoms feature bond lengths of 156.6(3) and 158.5(3) pm with an overall C1–F1–C2 bond angle of 115.78(15)°. This is consistent with the data of the computed quantum-chemical structure of cation **1** with C–F bond distances of 157.4 and 160.1 pm and a C–F–C angle of 115.32° (B3LYP/cc-pVTZ). Compared to the unsymmetrical bridging fluorine atom in Gabbaï’s bis-naphthalene complex with C–F distances of 142.4 and 244.4 pm, the distances in cation **1** are in between^13^. No interaction between anion and cation can be observed, although as predicted in previous publications, a single SbF_5_ coordinates to the anhydride function of the cation. The coordinating SbF_5_ is slightly bent out of the anhydride plane with a dihedral angle < (O2–C14–O1–Sb1) = 19.0(4)°, resulting in a *C*_1_ symmetry of the cation. Lectka et al. previously assumed *C*_s_ symmetry from their NMR analysis of this compound^16,17^.

**Fig. 3:**
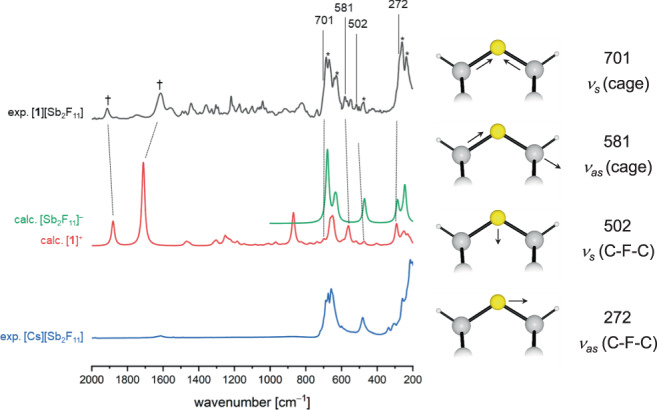
Vibrational analysis of fluoronium 1. Left: Experimental infrared spectra of [**1**][Sb_2_F_11_] (black) and [Cs][Sb_2_F_11_] (blue) at −40 °C, as well as calculated spectra of cation **1** (red) and anion [Sb_2_F_11_]^−^ (green) at B3LYP/def2-TZVPP level of theory. Bands of the anion are denoted with an asterisk in the experimental spectrum and bands associated with the carbonyl group are denoted with a dagger. Right: Approximate representation and assignment of selected C–F–C normal modes of **1** (see text, only displacements involving the C–F–C unit are shown).

The vacuum-dried crystalline material was investigated by IR spectroscopy at −40 °C. The experimental spectrum of [**1**][Sb_2_F_11_] (see Fig. 3, black trace) was assigned guided by calculated vibrational spectra of cation **1** and anion [Sb_2_F_11_]^−^ and by comparison with the spectrum of precursor **2** (Supplementary Fig. 3 and Supplementary Table 5), and an experimental spectrum of [Cs][Sb_2_F_11_]. This allows the assignment of characteristic vibrational modes at 581 cm^−1^ (calc. 560 cm^−1^) and 502 cm^−1^ (calc. 471 cm^−1^) carrying significant in-plane C–F–C stretching character. Although the region around 690 cm^−1^ and 260 cm^−1^ are dominated by strong Sb_2_F_11_ vibrations two additionally fluorine-involved modes are tentatively assigned to shoulders at 272 cm^−1^ (calc. 291 cm^−1^) and 701 cm^−1^ (calc. 699 cm^−1^). In addition, the coordination of SbF_5_ to one of the carbonyl groups of **1** leads to a splitting of the C=O bands at 1913 (ν(C=O)) and 1614 (ν(C=O⋅⋅⋅SbF_5_)) cm^−1^ (marked by a dagger symbol in Fig. 3). For a complete list of recorded IR vibrations and their assignment see also the methods section.

In Table 1 we present a comparison of the C–F–C stretching modes for the acyclic dimethyl halonium ions [Me_2_X]^+^ (X = F, Cl, Br and I) with those of the *C*_2v_ symmetric double-norbornyl halonium ions [DNTX]^+^ (without coordination of an additional SbF_5_ group). Generally, such a comparison is hampered by the more complex and rigid cage shape of the [DNTX]^+^ derivatives, since the division of such cage vibrations into certain stretching and ring deformations is more arbitrary and the vibrational coupling between different cage vibrations is more serious than for the simple dimethyl analogs. In Fig. 3 and Table 1 we present a tentative assignment of characteristic vibrations of **1** and the symmetric [DNTX]^+^ ions, respectively. A closer look reveals that the C–X–C stretching coordinates of the [DNTX]^+^ ions contribute to several normal modes such as an in-phase and an out-of-phase cage vibration, denoted as *ν*_s_(cage) and *ν*_as_(cage) in Table 1 and Fig. 3, respectively, in which mainly the two carbon atoms vibrate along the C–X coordinates, as well as to two lower-lying modes which carry dominant halogen atom displacements, and which are thus denoted as *ν*_s_(C–X–C) and *ν*_as_(C–X–C) stretching vibrations (for more details see Supplementary Movie 1). As a consequence, the *ν*_s_(cage) frequencies change only slightly from *ν*_s_ = 724 cm^−1^ ([DNTF]^+^) to 709 cm^−1^ ([DNTI]^+^), while the *ν*_s_(C–X–C) stretching frequencies in the [DNTX]^+^ ions are strongly reduced from *ν*_s_ = 488 cm^−1^ for X = F down to 365 cm^−1^ for X = I (Table 1).

**Table 1:** Comparison of selected computed vibrational frequencies and C–X–C bond angles of dimethyl halonium ions [Me_2_X]^+^ (X = F, Cl, Br, I) and double-norbornyl type halonium ions [DNTX]^+^ (*C*_2v_ symmetry) at def2-TZVPP/B3LYP level of theory.

		*ν*_s_ (cage)	*ν*_s_ (C–X–C)	*ν*_as_ (cage)	*ν*_as_ (C–X–C)	<(C–X–C)
	[DNTF]^+^	724 (6)	488 (6)	588 (29)	304 (34)	115°
	[DNTCl]^+^	711 (11)	386 (4)	657 (22)	313 (3)	98°
	[DNTBr]^+^	710 (15)	368 (4)	651 (19)	253 (2)	92°
	[DNTI]^+^	709 (18)	365 (3)	649 (14)	234 (1)	86°

		*ν*_s_ (C–X–C)	*δ* (C–X–C)	*ν*_as_ (C–X–C)		<(C–X–C)
	[Me_2_F]^+^	659 (12)	264 (1)	677 (100)		121°
	[Me_2_Cl]^+^	561 (41)	228 (1)	604 (100)		105°
	[Me_2_Br]^+^	500 (55)	187 (4)	517 (100)		101°
	[Me_2_I]^+^	470 (41)	160 (3)	484 (52)		98°

Frequencies are given in cm^−1^. Relative intensities are given in brackets.

The C–F–C stretching vibrations of **1** and the symmetric [DNTF]^+^ cation are strongly red-shifted compared to conventional monovalent C–F vibrations (usually observed between 1300 and 900 cm^−1^)^24^, indicating the weakened C–F bonds in these fluoronium derivatives. This is in line with similar findings of Dopfer et al. and their calculations on phenylfluoronium [*F*–C_6_H_6_F]^+^ ^25^. Also, the significantly higher frequency of the symmetric compared to the antisymmetric C–F–C stretching modes for the [DNTX]^+^ cations is remarkable and in striking contrast to spectroscopic investigations of acyclic dialkyl halonium salts^26,27^.

In addition, the comparison of the frequencies of the cyclic [DNTX]^+^ ions with those of the [Me_2_X]^+^ derivatives reveals further interesting features: while the *ν*_as_(C–X–C) vibrations of the [DNTX]^+^ ions generally occur at lower frequencies than those of the [Me_2_X]^+^ analogs, the two antisymmetric vibrations of [DNTF]^+^ are even lower in frequency than those of [DNTCl]^+^ (Table 1). We tentatively attribute these characteristic spectroscopic properties to different bonding properties of the C–X–C bonds in [DNTF]^+^ and [Me_2_F]^+^ on the one hand and the [DNTCl]^+^ cation on the other. A distortion along the antisymmetric stretching coordinate is expected to change the overall wave-function by increasing the relative weight of a carbo-cationic resonance structure with unequal C–F–C bond distances, and, consequently, the stabilization of this carbo-cationic resonance structure by suitable carbon substituents in a fluoronium equilibrium structure should result in a lower antisymmetric stretching frequency. This assumption was previously supported by an almost linear decrease in *ν*_as_(C–Cl–C) on the number of methyl groups *n* in the acyclic chloronium cations [(H_3_C)_*n*_(H_3−*n*_C)_2_Cl]^+^ (*n* = 0–3)^27^. Thus, lower *ν*_as_(C–X–C) frequencies are to be expected for the [DNTX]^+^ cations with secondary carbon substituents compared to the [Me_2_X]^+^ series. Also, entropic effects likely contribute to the stabilization of the cyclic [DNTX]^+^ cations. In addition, we have carried out a vibrational analysis of [DNTX]^+^ cations, with X = F and Cl, in which the hydrogen atoms of the H–C groups next to X are substituted by R = F, CH_3_, and CF_3_ (denoted as [R_2_DNTX]^+^ in Supplementary Table 4). With the exception of the fluorinated derivative [F_2_DNTF]^+^ (R = F) all other computed derivatives form stable symmetric halonium cations and for [F_2_DNTF]^+^, we have analyzed the *C*_2*v*_ symmetric fluoronium transition state, connecting two equivalent carbo-cationic minimum structures (Supplementary Fig. 2). For R = CH_3_ and F, which both support an asymmetric carbo-cationic structure the fluoronium ions (X = F) show lower ν_as_(C–X–C) frequencies than the chloronium analogs (X = Cl), which is in line with the above assumption. In contrast, the trifluoromethylated derivatives (R = CF_3_), which disfavors an ionic structure, show similar *ν*_as_(C–X–C) frequencies for X = F and Cl (Supplementary Table 4).

### Bonding analysis

Previous quantum-chemical studies focused on the atomic or partial charge of the fluorine atom in order to contest its classification as a fluoronium ion^28^. Atomic charges, however, strongly depend on the computational level and are not uniquely defined^29^. In the present case, a non-exhaustive selection of population analyses yields atomic charges for the bridging fluorine atom of −0.260 (NBO; all charges are given in atomic units), −0.136 (Mulliken), −0.132 (CHELPG), −0.521 (AIM), −0.094 (Merz–Kollmann), +0.058 (Voronoi) (for more details see Supplementary Table 6). For all methods, the neighboring carbon atoms yield a positive partial charge.

Perhaps a more relevant aspect is how the fluorine atom is bound to its two neighboring *sp*^3^-carbon atoms. As pointed out elsewhere, an AIM analysis shows two bond critical points (BCPs), indicating a chemical bond^16,17^. Judging from the different properties at these BCPs (*ρ*_BCP_ = 0.95 Å ^−3^; ∇^2^*ρ*_BCP_ = −6.43 Å^−5^; ELF_BCP_ = 0.43; |*V*|/*G* = 2.05) the bonds are barely covalent due to the strong fluorine-specific repulsion between lone pairs of the fluorine atom and the C–F *σ*-bonds and are best described as charge shift bonds^30,31^. This bond character differs significantly from the one in [H–F–H]^+^ (*ρ*_BCP_ = 2.03 Å^−3^; ∇^2^ρ_BCP_ = −68.44 Å^−5^; ELF_BCP_ = 0.98; |*V*|/*G* = 16.44), which is genuinely covalent^32^.

To compare cation **1** to its heavier analogs, the fluorine atom was replaced by other halogens. The positions of the halogen atoms, the two neighboring carbon atoms, and the two nearest hydrogen atoms were re-optimized, while all other atoms were kept fixed. Table 2 lists the most important properties of the BCPs in these four systems and in [H–F–H]^+^.

**Table 2:** Computed properties of the bonds to the halogen atom in different double-norbornyl type halonium ions: bond length (*r*_X–C_); deviation of the BCP from the mid-point of the bond (*r*_BCP–X_ − ½*r*_X–C_; for negative values, the BCP is closer to the halogen atom, for positive values, vice versa); electron density at the BCP (*ρ*_BCP_); Laplacian at the BCP (∇^2^*ρ*_BCP_); ELF at the BCP (ELF_BCP_); value of the ELF maximum along the bond path (ELF_max_); ratio of the absolute potential and the kinetic energy density at the BCP (|*V*|/*G*); localization index of the valence electrons at the halogen atom (^val^LI_X_); delocalization index of the bonds with the halogen atom (DLI_X–C_); localization index of the valence electrons at the carbon or hydrogen atom bound to the halogen atom (^val^LI_C/H_).

System	*r*_X–C_ [Å]	*r*_BCP–X_ − 1/2*r*_X–C_ [Å]	*ρ*_BCP_ [Å^−3^]	∇^2^*ρ*_BCP_ [Å^−5^]	ELF_BCP_	ELF_max_	|*V*|/*G*	^val^LI_X_	DLI_X–C_	^val^LI_C/H_
Fluoronium	1.5871	0.20	0.946	−6.432	0.43	–	2.05	6.72	0.58	1.84
Chloronium	1.8852	0.17	0.964	−2.614	0.80	0.87	2.49	5.82	0.85	1.91
Bromonium	2.0236	0.16	0.849	−1.616	0.82	0.83	2.40	4.73	0.89	1.95
Iodonium	2.2006	0.14	0.729	−1.099	0.80	0.82	2.33	5.32	0.94	2.03
[H–F–H]^+^	0.9679	0.35	2.027	−68.62	0.98	–	16.5	7.42	0.27	0.01

**Fig. 4:**
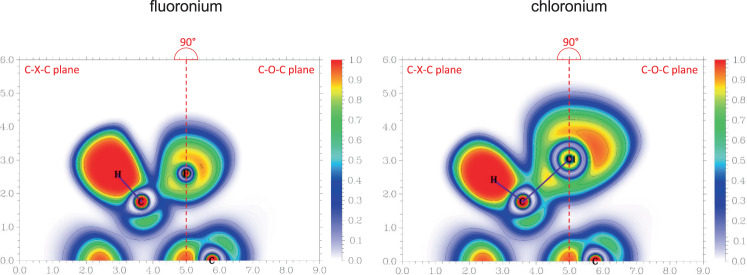
Electron localization in Fluoro- and Chloronium. Electron localization function in the C–X–C plane (X = F, Cl) of 1 (left) and its chloronium analog (right) and in their C–O–C planes containing the halogen’s lone pairs, perpendicular to the former one. Both planes are merged at the molecule’s O–X axis (dashed red line). ELF is defined from 0.0 (white) to 1.0 (red); contours are drawn in intervals of 0.1.

As the X–C bond distance increases the X–C bond becomes less polarized and the BCP approaches the mid-point of the X–C bond path. With increasing bond length, the electron density and its curvature at the BCP decreases, although the number of electrons associated with this bond increases, which can be seen from raising ELF (electron localization function) values and delocalization indices DLI_X–C_. The covalent character in the chlorine analog is slightly larger than in the fluoronium cation and decreases again for the bromonium and iodonium cation. Nevertheless, it never reaches values typical for genuine covalent bonds as in [H–F–H]^+^. In Fig. 4, ELF maps for the fluoronium and chloronium cations are shown for the C–X–C plane (X = F, Cl; left) and the one perpendicular to that containing the halogen lone pairs (right; bromonium and iodonium ELF maps in Supplementary Fig. 4). All four systems clearly indicate covalent interactions between carbon and the halogen atom, with the fluoronium cation resembling the least genuine covalent interaction and the chloronium cation the most. In the former, the valence electrons of the fluorine atom seem the least polarized, resembling almost the ELF map of an ion. This might be reinforced by the adjacent hydrogen atoms that draw electron density from the lone pair region in the C–F–C plane, which can be considered as a fluorine-specific interaction. For the other halonium cations, the valence shell is clearly separated into a maximum along the C–X bond path and two distinguished lone pairs.

In all, our results—loosely analogous to the reported norbornyl cation crystal structure in 2013^33^—definitively verify the nearly symmetrical structure of a controversial and often regarded as “impossible” species.

## Methods

### General considerations

All preparative work was carried out using standard Schlenk techniques. Glassware was greased with Triboflon III. All solid materials were handled inside a glove box with an atmosphere of dry argon (O_2_ < 0.5 ppm, H_2_O < 0.5 ppm). SO_2_ClF was stored over CaH_2_ before use. Precursor **2**, SbF_5_⋅SO_2_ and CsSb_2_F_11_ were made as reported^16,17,34,35^.

### IR

Low-temperature IR spectra were recorded on a Nicolet iS50 with a diamond ATR attachment and a home-build contraption which was cooled by a stream of liquid nitrogen to −40 °C.

### X-ray crystallography

All data were recorded on a Bruker D8 Venture diffractometer with a CMOS area detector using a Mo*K*α radiation source. In a nitrogen atmosphere suitable single crystals were coated and picked in perfluoroether oil at −80 °C and subsequently mounted on a 0.15 mm Micromount. The structure solution and refinement were performed in OLEX2^36^ utilizing the ShelXT^37^ structure solution program with intrinsic phasing and the ShelXL^38^ refinement package using least-squares on weighted F2 values for all reflections.

### Computational details

Calculations were performed with the Gaussian^39^ program, using the B3LYP DFT functional applying Dunnings cc-pVTZ basis set^40–42^ for all atoms except iodine for which a fully relativistic pseudopotential replacing 28 core electrons and the corresponding triple-ζ basis set^43,44^ was used. All population analyses, AIM analysis, as well as the calculation and visualization of ELF plots, were performed with the Multiwfn program^45^. In addition, the Turbomole program^46^ was used to perform calculations at the unrestricted Kohn–Sham DFT level, using the B3LYP hybrid functional^47–49^ in conjunction with the valence triple-ζ basis set with two sets of polarization functions (def2-TZVPP)^50^.

### Crystallization of fluoronium salt [1][Sb_2_F_11_]

After SbF_5_⋅SO_2_ (26 mg, 0.093 mmol) was filled into a Schlenk tube, SO_2_ClF was condensed into the vessel forming a clear solution at −50 °C. Precursor **2** (20 mg, 0.075 mmol) was added via a funnel to the reaction vessel and the mixture was shaken until a homogenous yellow solution was formed. After the partial evacuation of the solvent, the mixture was slowly cooled to −80 °C. Over the course of two weeks, yellow crystals started to grow which could be analyzed via X-ray diffraction.

Pumping off all volatiles from the crystalline material under reduced pressure produced an orange powder, of which a sample for low-temperature IR measurements was prepared.

IR (ATR, −40 °C): $\,\widetilde{\nu }$ = 2990 (w, *ν* (C–H)), 2914 (w, *ν* (C–H)), 1913 (m, *ν* (C=O)), 1746 (w), 1614 (s, *ν*_as_(C=O⋅⋅⋅Sb)), 1562 (m), 1492 (w), 1475 (w), 1443 (m), 1394 (w), 1359 (m), 1327 (w), 1304 (m), 1292 (w), 1275 (w), 1258 (w), 1218 (m), 1172 (m), 1136 (w), 1100 (m), 1068 (w), 1056 (w), 1042 (w), 1025 (w), 988 (w), 918 (w), 870 (w), 822 (m, *ν*_as_(C–O–C)), 793 (w), 736 (w), 701 (sh, *ν*_*s*_(C–F–C)), 685 (vs, *ν*(Sb–F)), 669 (vs, *ν*(Sb–F)), 640 (s, *ν*(Sb–F)), 629 (s, *ν*(Sb–F)), 581(s, *ν*_as_(C–F–C)), 551 (m), 516 (m, δ(C–F–C)), 502 (m), 476 (m, *ν* (Sb–F–Sb)), 430 (w), 417 (w), 408 (w), 384 (w), 364 (w), 272 (sh, *ρ* (C–F–C)), 237 (vs, δ(Sb–F–Sb)), 181 (w), 172 (w) cm^−1^. (vs = very strong, s = strong, m = medium, w = weak, sh = shoulder).

## Supplementary information

Supplementary Information

Supplementary Movie 1

Supplementary Dataset 1

Article File - Editor’s Summary

## Data Availability

Crystallographic data (excluding structure factors) for structures reported in this study have been deposited at the Cambridge Crystallographic Data Centre (CCDC) and can be obtained free of charge via www.ccdc.cam.ac.uk/data_request/cif on quoting the depository number CCDC-2049161. All other data generated or analyzed during this study are provided in this Article and the Supplementary Information.
