# Supplementary material for: Structural proof of a [C–F–C]+ fluoronium cation
Source: Nat Commun. 2021 Sep 6;12:5275. doi: 10.1038/s41467-021-25592-6 (PMC8421340; doi:10.1038/s41467-021-25592-6)
Supplement: Supplementary file 1 — Supplementary Information [file 41467_2021_25592_MOESM1_ESM.pdf]

Structural Proof of a [C–F–C]$^+$ Fluoronium Cation

Kurt F. Hoffmann, Anja Wiesner, Carsten Müller, Simon Steinhauer, Muhammad Kazim, Cody Ross Pitts, Thomas Lectka[2*], Sebastian Riedel[1*]

Correspondence to: [1] s.riedel@fu-berlin.de; [2] lectka@jhu.edu

Supplementary Information:

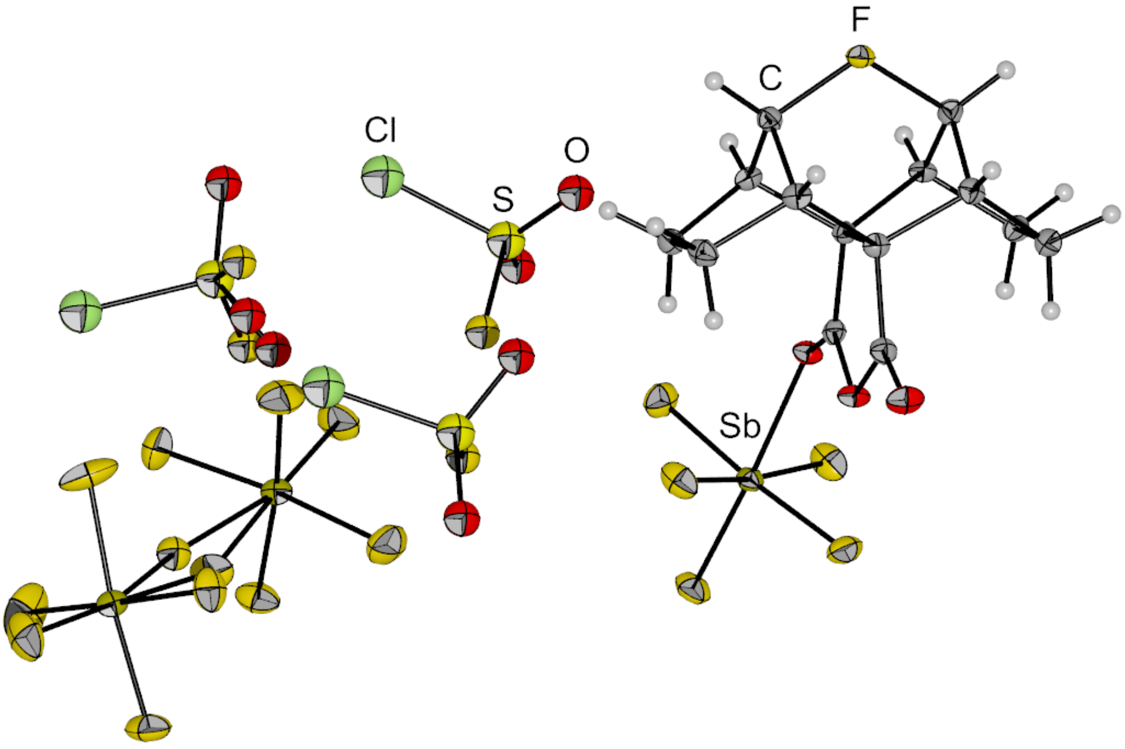

Supplementary Fig. 1. Molecular structure of [1][Sb_2F_{11}]·(SO_2ClF)$_3$. Thermal ellipsoids set at 50 % probability.

Supplementary Table 1. Summary of crystal data and refinement results

	**[1][Sb_2F_{11}]·(SO_2ClF)$_3$**
CCDC number	CCDC-2049161
empirical formula	$C_{14}H_{14}Cl_3F_{20}O_9S_3Sb_3$
formula weight	1274.03
temperature [K]	100.0
crystal system	monoclinic
space group	$P2_1/c$
a [pm]	9.4660(4)
b [pm]	25.0063(11)
c [pm]	14.8266(7)
α [°]	90
β [°]	92.532(2)
γ [°]	90
volume [Å^3]	3506.2(3)
Z	4
ρ_{calcd} [g · cm^{-3}]	2.414
μ [mm^{-1}]	2.850
dimension [mm]	0.435 × 0.185 × 0.164
color	yellow
reflection collected	101280
independent reflections	8747 [R_{int} = 0.0981]
data/restraints/parameters	8747/29/498
goodness-of-fit on F^2	1.040
final *R* indexes [$I > 2\sigma(I)$]	R_1 = 0.0328<br>wR_2 = 0.0549
final *R* indexes [all data]	R_1 = 0.0562<br>wR_2 = 0.0579

Supplementary Table 2. Bond lengths of [1][Sb_2F_{11}]·(SO_2ClF)$_3$.

Atom	Atom	Length/Å	Atom	Atom	Length/Å	Atom	Atom	Length/Å
Sb1	F2	1.8611(16)	Sb2	F12A	2.11(3)	O2	C13	1.495(3)
Sb1	F6	1.8613(17)	S1S	Cl1S	1.9727(12)	O3	C13	1.164(4)
Sb1	F5	1.8564(18)	S1S	F1S	1.527(2)	C14	C7	1.497(4)
Sb1	F4	1.8625(17)	S1S	O2S	1.405(3)	C7	C6	1.556(4)
Sb1	O1	2.0971(18)	S1S	O1S	1.400(2)	C7	C12	1.561(4)
Sb1	F3	1.8628(18)	S2S	Cl2S	1.9713(11)	C7	C8	1.546(4)
Sb3	F17	1.8489(18)	S2S	F2S	1.538(2)	C11	C10	1.546(4)
Sb3	F12	2.027(10)	S2S	O4S	1.401(2)	C11	C1	1.516(4)
Sb3	F16	1.8548(18)	S2S	O3S	1.398(3)	C11	C12	1.549(4)
Sb3	F13	1.854(2)	S3S	Cl3S	1.9565(17)	C10	C9	1.560(4)
Sb3	F14	1.850(2)	S3S	O5S	1.387(3)	C6	C2	1.517(4)
Sb3	F15	1.8535(19)	S3S	F3S	1.709(9)	C6	C5	1.548(4)
Sb3	F12A	1.91(4)	S3S	O6S	1.323(7)	C1	C8	1.524(4)
Sb2	F10	1.8589(17)	S3S	F3AS	1.526(7)	C12	C13	1.506(4)
Sb2	F11	1.8558(19)	S3S	O6AS	1.360(7)	C12	C3	1.553(4)
Sb2	F12	2.033(6)	F1	C1	1.566(3)	C8	C9	1.546(4)
Sb2	F9	1.851(2)	F1	C2	1.587(3)	C4	C3	1.548(4)
Sb2	F7	1.8469(19)	O1	C14	1.243(3)	C4	C5	1.568(4)
Sb2	F8	1.8554(18)	O2	C14	1.306(3)	C2	C3	1.511(4)

Supplementary Table 3. Bond angles of [1][Sb_2F_{11}]·(SO_2ClF)$_3$.

Atom	Atom	Atom	Angle/°	Atom	Atom	Atom	Angle/°	Atom	Atom	Atom	Angle/°
F2	Sb1	F6	94.66(8)	F7	Sb2	F12	178.0(4)	C1	F1	C2	115.64(17)
F2	Sb1	F4	93.88(8)	F7	Sb2	F9	94.03(10)	C14	O1	Sb1	129.82(18)
F2	Sb1	O1	176.97(8)	F7	Sb2	F8	95.15(9)	Sb3	F12	Sb2	155.9(7)
F2	Sb1	F3	91.80(8)	F7	Sb2	F12A	165(5)	C14	O2	C13	110.7(2)
F6	Sb1	F4	171.46(7)	F8	Sb2	F10	169.22(9)	O1	C14	O2	122.5(2)
F6	Sb1	O1	86.01(7)	F8	Sb2	F11	88.30(9)	O1	C14	C7	123.8(3)
F6	Sb1	F3	89.63(9)	F8	Sb2	F12	85.95(17)	O2	C14	C7	113.7(2)
F5	Sb1	F2	92.88(8)	F8	Sb2	F12A	84.6(19)	C14	C7	C6	113.7(2)
F5	Sb1	F6	89.48(9)	F1S	S1S	Cl1S	98.88(11)	C14	C7	C12	103.6(2)
F5	Sb1	F4	90.01(8)	O2S	S1S	Cl1S	109.41(12)	C14	C7	C8	116.8(2)
F5	Sb1	O1	84.16(8)	O2S	S1S	F1S	106.94(15)	C6	C7	C12	104.1(2)
F5	Sb1	F3	175.29(8)	O1S	S1S	Cl1S	108.97(13)	C8	C7	C6	112.4(2)
F4	Sb1	O1	85.46(7)	O1S	S1S	F1S	107.82(16)	C8	C7	C12	104.6(2)
F4	Sb1	F3	90.18(9)	O1S	S1S	O2S	122.26(17)	C10	C11	C12	110.7(2)
F3	Sb1	O1	91.16(8)	F2S	S2S	Cl2S	98.17(9)	C1	C11	C10	97.8(2)
F17	Sb3	F12	84.9(5)	O4S	S2S	Cl2S	108.52(11)	C1	C11	C12	99.5(2)
F17	Sb3	F16	90.58(9)	O4S	S2S	F2S	107.11(14)	C11	C10	C9	104.2(2)
F17	Sb3	F13	92.90(9)	O3S	S2S	Cl2S	110.33(12)	C2	C6	C7	99.0(2)
F17	Sb3	F14	172.59(11)	O3S	S2S	F2S	107.18(15)	C2	C6	C5	97.1(2)
F17	Sb3	F15	89.84(10)	O3S	S2S	O4S	122.68(16)	C5	C6	C7	111.2(2)
F17	Sb3	F12A	99(3)	O5S	S3S	Cl3S	109.55(14)	C11	C1	F1	109.5(2)
F16	Sb3	F12	84.13(18)	O5S	S3S	F3S	93.3(4)	C11	C1	C8	99.2(2)
F16	Sb3	F12A	95(4)	O5S	S3S	F3AS	114.6(4)	C8	C1	F1	108.1(2)
F13	Sb3	F12	177.0(5)	F3S	S3S	Cl3S	97.9(3)	C11	C12	C7	103.5(2)
F13	Sb3	F16	93.85(10)	O6S	S3S	Cl3S	116.6(4)	C11	C12	C3	112.6(2)
F13	Sb3	F12A	165(5)	O6S	S3S	O5S	128.6(4)	C13	C12	C7	105.3(2)
F14	Sb3	F12	87.7(5)	O6S	S3S	F3S	101.4(5)	C13	C12	C11	115.2(2)
F14	Sb3	F16	89.06(10)	F3AS	S3S	Cl3S	101.0(3)	C13	C12	C3	114.5(2)
F14	Sb3	F13	94.51(11)	O6AS	S3S	Cl3S	99.3(4)	C3	C12	C7	104.1(2)
F14	Sb3	F15	89.35(11)	O6AS	S3S	O5S	121.8(5)	C7	C8	C9	110.7(2)
F14	Sb3	F12A	74(3)	O6AS	S3S	F3AS	107.7(5)	C1	C8	C7	99.1(2)
F15	Sb3	F12	86.81(19)	O4S	S2S	Cl2S	108.52(11)	C1	C8	C9	97.5(2)
F15	Sb3	F16	170.85(10)	O4S	S2S	F2S	107.11(14)	C3	C4	C5	103.9(2)
F15	Sb3	F13	95.26(10)	O3S	S2S	Cl2S	110.33(12)	C8	C9	C10	104.0(2)
F15	Sb3	F12A	76(4)	O3S	S2S	F2S	107.18(15)	O2	C13	C12	106.7(2)
F10	Sb2	F12	83.45(18)	O3S	S2S	O4S	122.68(16)	O3	C13	O2	118.0(2)
F10	Sb2	F12A	85(2)	O5S	S3S	Cl3S	109.55(14)	O3	C13	C12	135.3(3)
F11	Sb2	F10	89.23(9)	O5S	S3S	F3S	93.3(4)	C6	C2	F1	108.4(2)
F11	Sb2	F12	87.3(5)	O5S	S3S	F3AS	114.6(4)	C3	C2	F1	108.4(2)
F11	Sb2	F12A	70(5)	F3S	S3S	Cl3S	97.9(3)	C3	C2	C6	99.9(2)
F9	Sb2	F10	90.18(9)	O6S	S3S	Cl3S	116.6(4)	C4	C3	C12	111.3(2)
F9	Sb2	F11	171.47(10)	O6S	S3S	O5S	128.6(4)	C2	C3	C12	99.5(2)
F9	Sb2	F12	84.2(5)	O6S	S3S	F3S	101.4(5)	C2	C3	C4	97.1(2)
F9	Sb2	F8	90.71(10)	F3AS	S3S	Cl3S	101.0(3)	C6	C5	C4	104.2(2)
F9	Sb2	F12A	101(5)	O6AS	S3S	Cl3S	99.3(4)	Sb3	F12A	Sb2	162(6)
F7	Sb2	F10	95.51(9)	O6AS	S3S	O5S	121.8(5)				
F7	Sb2	F11	94.49(10)	O6AS	S3S	F3AS	107.7(5)				

Supplementary Table 4. Calculated vibrational frequencies and C–X–C bond angles of different dimethyl halonium ions $[Me_2X]^+$ (X = F, Cl, Br, I), double-norbornyl type halonium ions $[DNTX]^+$ (C_{2v} symmetry) and HC-substituted $[R_2DNTX]^+$ halonium ions (R = F, Me, CF_3) at def2-TZVPP/B3LYP level of theory. Frequencies are given in cm^{-1}. Relative intensities are given in brackets.

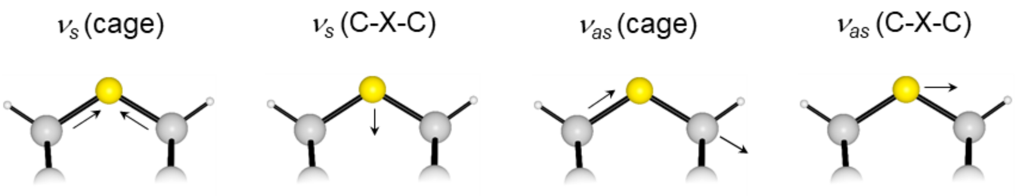

		ν_s (cage)	ν_s (C-X-C)	ν_{as} (cage)	ν_{as} (C-X-C)	<(C-X-C)
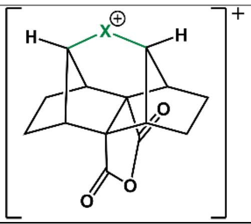	$[DNTF]^+$	724 (6)	488 (6)	588 (29)	304 (34)	115°
	$[DNTCl]^+$	711 (11)	386 (4)	657 (22)	313 (3)	98°
	$[DNTBr]^+$	710 (15)	368 (4)	651 (19)	253 (2)	92°
	$[DNTI]^+$	709 (18)	365 (3)	649 (14)	234 (1)	86°
		ν_s (C-X-C)	δ (C-X-C)	ν_{as} (C-X-C)		<(C-X-C)
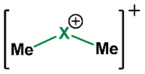	$[Me_2F]^+$	659 (12)	264 (1)	677 (100)		121°
	$[Me_2Cl]^+$	561 (41)	228 (1)	604 (100)		105°
	$[Me_2Br]^+$	500 (55)	187 (4)	517 (100)		101°
	$[Me_2I]^+$	470 (41)	160 (3)	484 (52)		98°
		ν_s (cage)	ν_s (C-X-C)	ν_{as} (cage)	ν_{as} (C-X-C)	<(C-X-C)
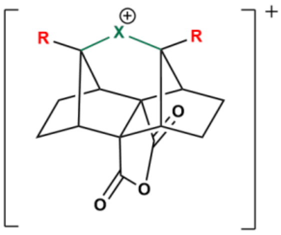	$[F_2DNTF]^{+}$ [a)]	850/800	552/474	688	-168	115°
	$[F_2DNTCl]^+$	831/780	519/415	685	237	97°
	$[(CH_3)_2DNTF]^+$	708/679	546/467	638/552	176[b)]	119°
	$[(CH_3)_2DNTCl]^+$	709/667	518/402	660/563	363[c)]	102°
	$[(CF_3)_2DNTF]^+$	847/803	509	691/537	375	118°
	$[(CF_3)_2DNTCl]^+$	868/792	405	715/546[d)]	376	101°

[a)]: Transition state connecting two carbo-cationic structure
[b)]: coupled with modes at 434, 341 and 161 cm^{-1}
[c)]: coupled with mode at 278 cm^{-1}
[d)]: coupled with modes at 764 and 742 cm^{-1}

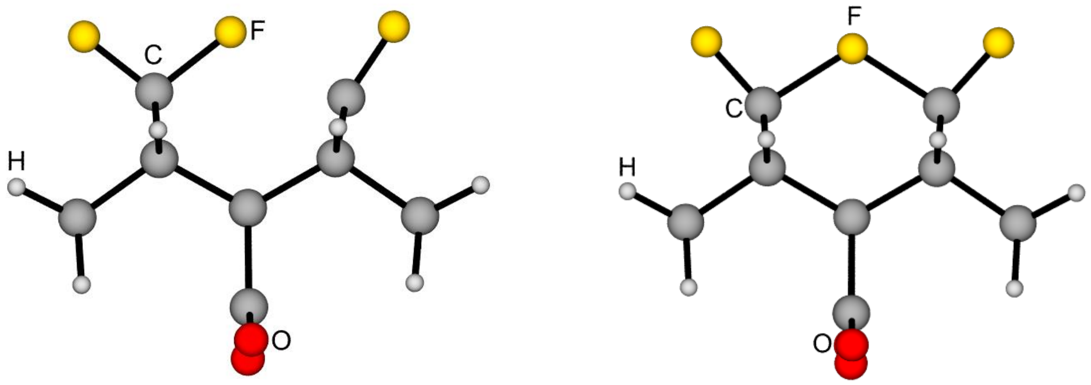

Supplementary Fig. 2. Calculated structures of $[F_2DNTF]^+$ (B3LYP/def2-TZVPP). Left: Ground state with carbo-cationic structure. Right: Transition state with C_{2v} symmetry.

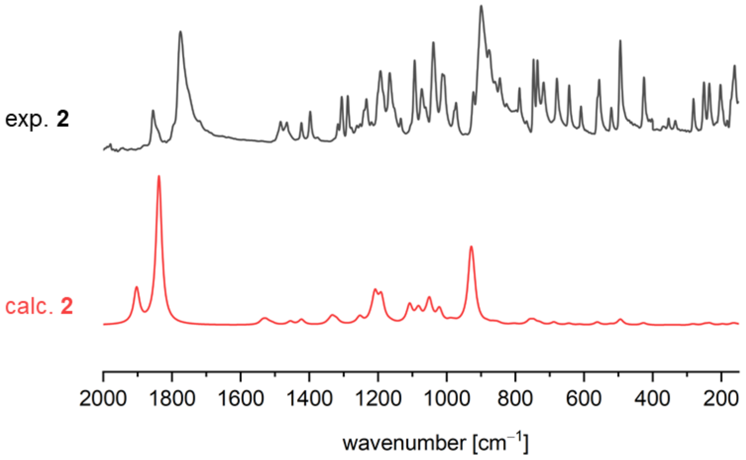

Supplementary Fig. 3. Experimental and calculated (B3LYP/def2-TZVPP) IR spectra of precursor **2**.

Supplementary Table 5. Selected experimental and calculated (B3LYP/def2-TZVPP) vibrations of precursor **2**.

Frequencies [cm^{-1}]		Assignments
experimental	calculated	
1855 (m)	1903 (m)	ν_s(C=O)
1775 (vs)	1838 (vs)	ν_{as}(C=O)
900 (vs)	930 (s)	ν_{as} (C-O-C)

vs=very strong, s=strong, m=medium

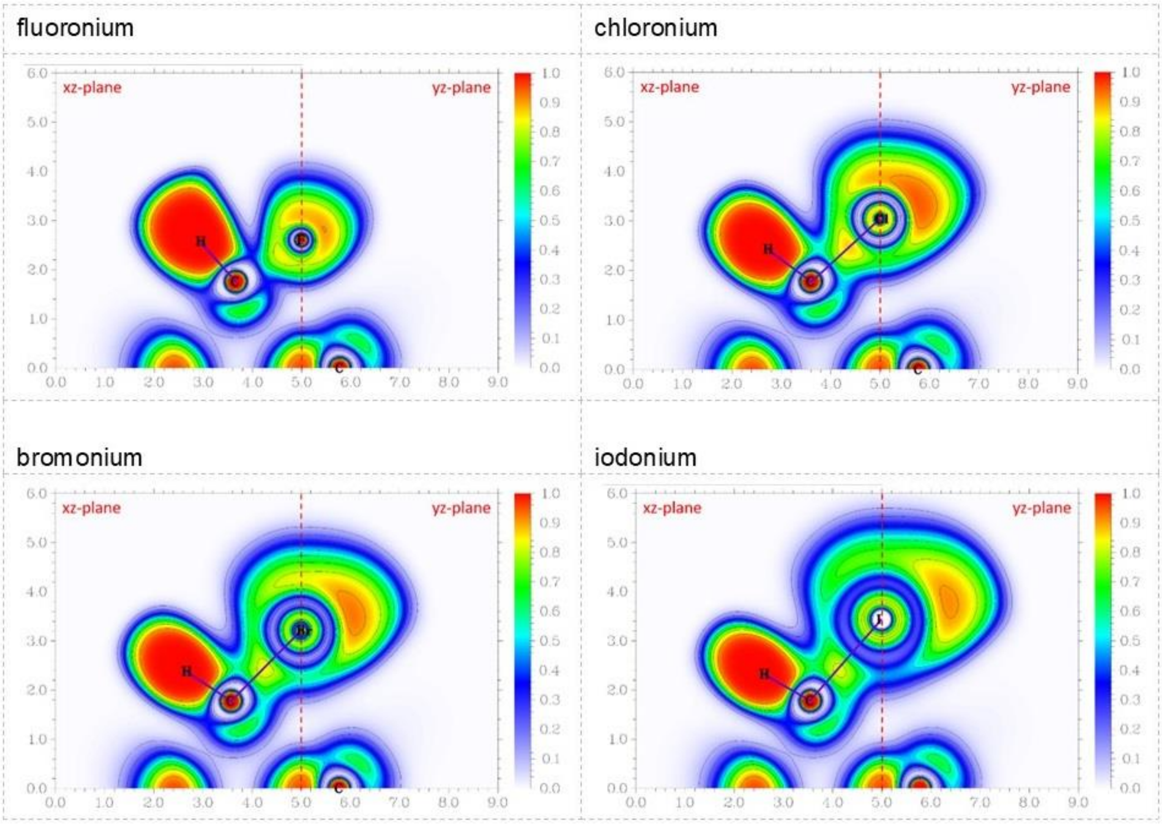

Supplementary Fig. 4. Electron localization function in the C–X–C plane (“xz-plane”) and the plane containing the halogen’s lone pairs, perpendicular to the former one (yz-plane). Both planes are merged at the molecule’s main axis (dashed red line). ELF is defined from 0.0 (white) to 1.0 (red); contours are drawn in intervals of 0.1.

Supplementary Table 6. Atomic charges of fluoronium in C_{2v} symmetry.

Atom(s)	AIM	NBO	Mulliken	ChelpG	Merz-Kollmann	Voronoi	Löwdin
1 F	-0.521	-0.260	-0.136	-0.132	-0.094	0.058	0.382
2 O	-0.031	-0.490	-0.163	-0.441	-0.480	-0.100	0.399
3/13 C	-0.009	-0.119	-0.063	0.038	-0.120	-0.031	-0.067
4/9/14/25 C	0.041	-0.256	-0.046	-0.219	-0.237	-0.042	0.029
5/10/15/26 H	0.072	0.254	0.139	0.128	0.174	0.091	0.005
6/16/19/27 C	0.000	-0.388	-0.223	-0.036	-0.101	-0.042	0.100
7/18/21/28 H	0.056	0.238	0.140	0.067	0.106	0.082	0.001
8/17/20/29 H	0.050	0.233	0.147	0.075	0.108	0.066	0.002
11/23 C	0.245	0.285	0.150	0.284	0.258	0.116	0.083
12/24 H	0.141	0.236	0.177	0.135	0.160	0.117	0.012
22/30 C	1.556	0.788	0.286	0.734	0.839	0.225	-0.453
31/32 O	-1.101	-0.479	-0.216	-0.444	-0.448	-0.217	0.259
