# Supplementary material for: Structural proof of a [C–F–C]+ fluoronium cation
Source: Nat Commun. 2021 Sep 6;12:5275. doi: 10.1038/s41467-021-25592-6 (PMC8421340; doi:10.1038/s41467-021-25592-6)
Supplement: Supplementary file 3 — Supplementary Dataset 1 [file 41467_2021_25592_MOESM3_ESM.docx]

**Supplementary Data Table 1.** Cartesian coordinates.

Cartesian coordinates of optimized structures for AIM analysis on cc-pVTZ/B3LYP level of theory:

**Fluoronium (*C*_2v_)**

1 9 0.000000 0.000000 2.583669

2 8 0.000000 0.000000 -2.263264

3 6 0.000000 0.781841 -0.026580

4 6 1.291134 -1.151111 0.753468

5 1 1.295331 -2.153093 1.170604

6 6 2.560805 -0.783278 -0.056647

7 1 2.532376 -1.217377 -1.053202

8 1 3.447342 -1.179173 0.434062

9 6 -1.291134 1.151111 0.753468

10 1 -1.295331 2.153093 1.170604

11 6 1.340081 0.000000 1.733264

12 1 2.049299 0.000000 2.552572

13 6 0.000000 -0.781841 -0.026580

14 6 1.291134 1.151111 0.753468

15 1 1.295331 2.153093 1.170604

16 6 -2.560805 -0.783278 -0.056647

17 1 -3.447342 -1.179173 0.434062

18 1 -2.532376 -1.217377 -1.053202

19 6 2.560805 0.783278 -0.056647

20 1 3.447342 1.179173 0.434062

21 1 2.532376 1.217377 -1.053202

22 6 0.000000 -1.160633 -1.495304

23 6 -1.340081 0.000000 1.733264

24 1 -2.049299 0.000000 2.552572

25 6 -1.291134 -1.151111 0.753468

26 1 -1.295331 -2.153093 1.170604

27 6 -2.560805 0.783278 -0.056647

28 1 -2.532376 1.217377 -1.053202

29 1 -3.447342 1.179173 0.434062

30 6 0.000000 1.160633 -1.495304

31 8 0.000000 -2.240491 -1.982386

32 8 0.000000 2.240491 -1.982386

**Chloronium (*C*_2v_)**

1 17 0.000000 -2.731549 0.000000

2 8 0.000000 2.549200 0.000000

3 6 0.000000 0.312500 -0.781800

4 6 -1.291100 -0.467500 1.151100

5 1 -1.295300 -0.884600 2.153100

6 6 -2.560800 0.342600 0.783300

7 1 -2.532400 1.339200 1.217400

8 1 -3.447300 -0.148100 1.179200

9 6 1.291100 -0.467500 -1.151100

10 1 1.295300 -0.884600 -2.153100

11 6 -1.397787 -1.466505 0.000000

12 1 -2.263869 -2.120155 0.000000

13 6 0.000000 0.312500 0.781800

14 6 -1.291100 -0.467500 -1.151100

15 1 -1.295300 -0.884600 -2.153100

16 6 2.560800 0.342600 0.783300

17 1 3.447300 -0.148100 1.179200

18 1 2.532400 1.339200 1.217400

19 6 -2.560800 0.342600 -0.783300

20 1 -3.447300 -0.148100 -1.179200

21 1 -2.532400 1.339200 -1.217400

22 6 0.000000 1.781300 1.160600

23 6 1.397787 -1.466505 0.000000

24 1 2.263869 -2.120155 0.000000

25 6 1.291100 -0.467500 1.151100

26 1 1.295300 -0.884600 2.153100

27 6 2.560800 0.342600 -0.783300

28 1 2.532400 1.339200 -1.217400

29 1 3.447300 -0.148100 -1.179200

30 6 0.000000 1.781300 -1.160600

31 8 0.000000 2.268300 2.240500

32 8 0.000000 2.268300 -2.240500

**Bromonium (*C*_2v_)**

1 35 0.000000 -2.902612 0.000000

2 8 0.000000 2.549200 0.000000

3 6 0.000000 0.312500 -0.781800

4 6 -1.291100 -0.467500 1.151100

5 1 -1.295300 -0.884600 2.153100

6 6 -2.560800 0.342600 0.783300

7 1 -2.532400 1.339200 1.217400

8 1 -3.447300 -0.148100 1.179200

9 6 1.291100 -0.467500 -1.151100

10 1 1.295300 -0.884600 -2.153100

11 6 -1.424617 -1.465524 0.000000

12 1 -2.324301 -2.071049 0.000000

13 6 0.000000 0.312500 0.781800

14 6 -1.291100 -0.467500 -1.151100

15 1 -1.295300 -0.884600 -2.153100

16 6 2.560800 0.342600 0.783300

17 1 3.447300 -0.148100 1.179200

18 1 2.532400 1.339200 1.217400

19 6 -2.560800 0.342600 -0.783300

20 1 -3.447300 -0.148100 -1.179200

21 1 -2.532400 1.339200 -1.217400

22 6 0.000000 1.781300 1.160600

23 6 1.424617 -1.465524 0.000000

24 1 2.324301 -2.071049 0.000000

25 6 1.291100 -0.467500 1.151100

26 1 1.295300 -0.884600 2.153100

27 6 2.560800 0.342600 -0.783300

28 1 2.532400 1.339200 -1.217400

29 1 3.447300 -0.148100 -1.179200

30 6 0.000000 1.781300 -1.160600

31 8 0.000000 2.268300 2.240500

32 8 0.000000 2.268300 -2.240500

**Iodonium (*C*_2v_)**

1 53 0.000000 0.000000 2.513071

2 8 0.000000 0.000000 -3.162435

3 6 0.000000 0.781800 -0.925735

4 6 1.291100 -1.151100 -0.145735

5 1 1.295300 -2.153100 0.271365

6 6 2.560800 -0.783300 -0.955835

7 1 2.532400 -1.217400 -1.952435

8 1 3.447300 -1.179200 -0.465135

9 6 -1.291100 1.151100 -0.145735

10 1 -1.295300 2.153100 0.271365

11 6 1.448222 0.000000 0.856152

12 1 2.385120 0.000000 1.401621

13 6 0.000000 -0.781800 -0.925735

14 6 1.291100 1.151100 -0.145735

15 1 1.295300 2.153100 0.271365

16 6 -2.560800 -0.783300 -0.955835

17 1 -3.447300 -1.179200 -0.465135

18 1 -2.532400 -1.217400 -1.952435

19 6 2.560800 0.783300 -0.955835

20 1 3.447300 1.179200 -0.465135

21 1 2.532400 1.217400 -1.952435

22 6 0.000000 -1.160600 -2.394535

23 6 -1.448222 0.000000 0.856152

24 1 -2.385120 0.000000 1.401621

25 6 -1.291100 -1.151100 -0.145735

26 1 -1.295300 -2.153100 0.271365

27 6 -2.560800 0.783300 -0.955835

28 1 -2.532400 1.217400 -1.952435

29 1 -3.447300 1.179200 -0.465135

30 6 0.000000 1.160600 -2.394535

31 8 0.000000 -2.240500 -2.881535

32 8 0.000000 2.240500 -2.881535

Cartesian coordinates of optimized structures on def2-TZVPP/B3LYP level of theory:

**Fluoronium [1]^+^**

$coord

11.09534536303368 28.13188835984827 14.98931574951190 sb

0.57216723090669 27.18635296840741 23.97693675753127 f

13.45970764792514 29.37169543806405 12.66314411910007 f

12.89869675354930 25.34190635722174 16.30878115398685 f

12.09698277036908 30.08465590272153 17.82438207564522 f

8.48036251955925 30.57788399531997 14.44804876626062 f

8.08177167933374 26.82694386471859 18.02505495499407 o

9.34066589251418 25.91085935379019 12.79127053555617 f

4.52061988878575 26.56302996220730 15.65722640554323 o

0.41113278846160 26.36550555200010 14.29478224964746 o

5.78132212388425 26.74627742697040 17.86817302069135 c

3.99550155498554 26.88659795507331 20.09253621459212 c

0.06286343958818 24.41190855002334 20.10573586467333 c

-2.00256669452309 24.38557524609745 20.03144500624721 h

1.35875178479745 21.93440946445377 19.20817075374301 c

1.39819224350847 21.81648614264561 17.13967932670655 h

0.29382714193859 20.28758632118030 19.87534246517582 h

4.01058707044167 29.46752030419488 21.51105958559663 c

5.41507262608422 29.60911305426804 23.02124085695728 h

1.27290479063495 24.54918693602359 22.70119959849152 c

0.63307960097336 23.30212823780058 24.21928585693571 h

1.30113896917773 26.76909683044017 18.85237041330019 c

4.03216740479310 24.58832335931228 21.92544025283144 c

5.43459036956572 24.72225006565212 23.43854940285828 h

1.30791392793588 31.60185939963047 18.38883428935081 c

0.22051961140715 33.32312531942887 18.77016712795976 h

1.35226794681177 31.37049788611540 16.32994868629854 h

4.05453080686021 22.05554586373663 20.44262096387240 c

4.36737729390755 20.47044804863183 21.73787402062116 h

5.60253257414828 22.00519103120543 19.06465760867153 h

1.80424062170627 26.54155988477370 16.03994744661705 c

1.25576295438694 29.61736689579349 22.26185907906203 c

0.59451265755745 31.07472635593292 23.56804894504331 h

0.03867448585722 29.29349207141757 19.69160857607504 c

-2.02704809660869 29.28298040929766 19.62039067964281 h

4.00534297198925 31.72041463847492 19.62270396968651 c

5.55225457735762 31.56520806418354 18.24995482487319 h

4.29496277014760 33.50037467875320 20.64117661561667 h

**[Sb_2_F_11_]^−^**

$coord

3.87268788333358 -0.09894696956553 0.00716070108642 sb

-3.87106180713710 0.09657821958757 -0.01277906084533 sb

3.18342454007231 1.65349371533709 3.03110677998590 f

-0.00223942088031 -0.18049990697928 -0.57843781185082 f

7.40868981086617 -0.03892792783807 0.49198105490099 f

4.08985798566229 -1.85886318631115 -3.08816893251563 f

-3.26604319661623 3.44691384990498 1.04002256439009 f

-7.40188892547857 0.33336050787399 0.45537915223984 f

-4.01199458317984 -3.28272924388573 -1.14461879401264 f

-3.26498416123545 -1.05426170745525 3.30421156245971 f

-4.00850489931800 1.20626944810937 -3.40002027407987 f

3.45338159547559 -3.20035256431438 1.71377775106013 f

3.82957426312473 2.98597174246114 -1.78070366486947 f

**Precursor 2**

$coord

0.41781573334380 25.43754234821739 24.88988623714344 f

7.95218255080806 26.79989990358887 18.04600445513265 o

4.29652909826633 26.61665200324481 15.88724251763853 o

0.22793878235309 26.46242005688439 14.66989844692075 o

5.70540658466113 26.79911951309763 18.10347670431808 c

3.93555706332755 26.96593916967636 20.35859227165888 c

0.11496912567343 24.34086981279938 20.27443076468609 c

-1.93464089090401 24.22468356832750 20.20784982173707 h

1.49957705643056 22.07436347852333 19.01254786481486 c

1.51386692173675 22.16979551375601 16.96122429239659 h

0.54981001828699 20.31818268432746 19.51177075899795 h

4.03494983113528 29.65198712300927 21.57712703252322 c

5.47825930121785 29.86118459734848 23.02435444424643 h

1.34813731823086 24.00653384820365 22.89199225647063 c

1.14580610375334 22.06373866617729 23.54387364535686 h

1.23819806641386 26.84999337283288 19.17937788516309 c

4.07479950162451 24.50919127616777 22.00217331826874 c

5.52081925778058 24.54022364202098 23.46111159202404 h

1.43166876934926 31.55421366392643 18.26243712386893 c

0.45271502187929 33.34308240129546 18.51312380074426 h

1.44390082316876 31.15563012338758 16.24590423475416 h

4.22275013303083 22.19231188548677 20.19850918598694 c

4.63010229477829 20.49301330935876 21.28590960723543 h

5.70601588656829 22.35448083949612 18.78774287662409 h

1.70554631404996 26.62493122475932 16.35537999422840 c

1.28298450773030 30.03788632974857 22.43788277287040 c

0.84725849783879 32.53773163636995 23.18231120145295 f

0.08466116536951 29.48246322768367 19.84685652068797 c

-1.96730049951306 29.54029968936661 19.76266484237666 h

4.13891351260622 31.67131540713741 19.44896659589750 c

5.64023093674998 31.33785529377679 18.08558868845336 h

4.48509881318468 33.51683563466127 20.28286951886025 h

0.65539711360971 28.88208428007490 23.98613949589301 h

**[DNTF]^+^**

$coord

0.62903568283625 27.17298445796316 23.92159109701894 f

8.07856945357527 26.85696481285739 17.84738964852043 o

4.45291128427226 26.61110531628079 15.61363070759538 o

0.38689764877892 26.42686067099202 14.33683571558249 o

5.84114881258226 26.81038949855044 17.83832040575475 c

4.03191218555459 26.94436673880458 20.06055962338056 c

0.14500924431308 24.41814258306388 20.06943202153789 c

-1.90513381121230 24.36362003198772 20.00038036324571 h

1.46346277527762 21.96613744302900 19.15426538343608 c

1.50115237499959 21.86046788654414 17.10163047313296 h

0.42971043231209 20.31541661339373 19.81530905013500 h

4.00082771774082 29.50325147452042 21.49965437443050 c

5.39213118763003 29.65591777224739 23.00073572766194 h

1.35103174846976 24.54966384364540 22.65879625422651 c

0.73208806491786 23.30736521101023 24.16579139583224 h

1.34504850554597 26.79566097638355 18.83368875036909 c

4.09924238190754 24.63652531330480 21.87326990345215 c

5.49134762142820 24.77228568383275 23.37520464073304 h

1.26966352713846 31.61909371204081 18.41123948692532 c

0.16888106131252 33.30854207217045 18.81617234948391 h

1.31002121765530 31.41183276272340 16.36650773461949 h

4.15408095867805 22.11573019261776 20.38039528269804 c

4.48278527604875 20.53964052669656 21.65940492915440 h

5.68442968815264 22.09530542666814 19.00733637624777 h

1.85444509625285 26.58826290881372 16.01853196108277 c

1.24893877316845 29.60011408074350 22.26880479938782 c

0.57519577846263 31.03145576062675 23.57042219627753 h

0.04704012358508 29.28455019730367 19.69374678160445 c

-2.00341485645549 29.24551578882527 19.62251615502302 h

3.96066521329003 31.76592173727553 19.63813571363634 c

5.49297076580742 31.63528648522188 18.27400065472762 h

4.22226124040187 33.53112970508958 20.66018358264897 h

**[DNTCl]^+^**

$coord

0.00000000000000 0.00000000000000 5.27106239521996 cl

0.00000000000000 0.00000000000000 -4.61318355979169 o

0.00000000000000 1.47487823363886 -0.37866368450989 c

2.47788421815234 -2.17540670325418 1.03860713823379 c

2.51036727783312 -4.07778110941794 1.80828946247499 h

4.81858109059850 -1.48023311220189 -0.58259715627306 c

4.70855408391570 -2.29664686845931 -2.46581124257644 h

6.52408028693769 -2.22903617860494 0.28956444298374 h

-2.47788421815234 2.17540670325418 1.03860713823379 c

-2.51036727783312 4.07778110941794 1.80828946247499 h

2.68559021570232 0.00000000000000 2.92676002870376 c

4.30480696789164 0.00000000000000 4.18850348554270 h

0.00000000000000 -1.47487823363886 -0.37866368450989 c

2.47788421815234 2.17540670325418 1.03860713823379 c

2.51036727783312 4.07778110941794 1.80828946247499 h

-4.81858109059850 -1.48023311220189 -0.58259715627306 c

-6.52408028693769 -2.22903617860494 0.28956444298374 h

-4.70855408391570 -2.29664686845931 -2.46581124257644 h

4.81858109059850 1.48023311220189 -0.58259715627306 c

6.52408028693769 2.22903617860494 0.28956444298374 h

4.70855408391570 2.29664686845931 -2.46581124257644 h

0.00000000000000 -2.19014356126564 -3.15967221246382 c

-2.68559021570232 0.00000000000000 2.92676002870376 c

-4.30480696789164 0.00000000000000 4.18850348554270 h

-2.47788421815234 -2.17540670325418 1.03860713823379 c

-2.51036727783312 -4.07778110941794 1.80828946247499 h

-4.81858109059850 1.48023311220189 -0.58259715627306 c

-4.70855408391570 2.29664686845931 -2.46581124257644 h

-6.52408028693769 2.22903617860494 0.28956444298374 h

0.00000000000000 2.19014356126564 -3.15967221246382 c

0.00000000000000 -4.23010334968507 -4.08197232467304 o

0.00000000000000 4.23010334968507 -4.08197232467304 o

**[DNTBr]^+^**

$coord

0.00000000000000 0.00000000000000 5.59854979991570 br

0.00000000000000 0.00000000000000 -4.57788273771324 o

0.00000000000000 1.47343681101759 -0.34009472067078 c

2.49537860177614 -2.17523898796772 1.05048688723752 c

2.54196487967722 -4.07984510626205 1.81324551866535 h

4.81038407749094 -1.48089318818146 -0.61581710583899 c

4.67016212073147 -2.29681294620007 -2.49730646214463 h

6.52941416286428 -2.22991847155006 0.22853500790013 h

-2.49537860177614 2.17523898796772 1.05048688723752 c

-2.54196487967722 4.07984510626205 1.81324551866535 h

2.76911866699108 0.00000000000000 2.93040297316913 c

4.44797190215980 0.00000000000000 4.10931739741494 h

0.00000000000000 -1.47343681101759 -0.34009472067078 c

2.49537860177614 2.17523898796772 1.05048688723752 c

2.54196487967722 4.07984510626205 1.81324551866535 h

-4.81038407749094 -1.48089318818146 -0.61581710583899 c

-6.52941416286428 -2.22991847155006 0.22853500790013 h

-4.67016212073147 -2.29681294620007 -2.49730646214463 h

4.81038407749094 1.48089318818146 -0.61581710583899 c

6.52941416286428 2.22991847155006 0.22853500790013 h

4.67016212073147 2.29681294620007 -2.49730646214463 h

0.00000000000000 -2.18884024376465 -3.12288654687132 c

-2.76911866699108 0.00000000000000 2.93040297316913 c

-4.44797190215980 0.00000000000000 4.10931739741494 h

-2.49537860177614 -2.17523898796772 1.05048688723752 c

-2.54196487967722 -4.07984510626205 1.81324551866535 h

-4.81038407749094 1.48089318818146 -0.61581710583899 c

-4.67016212073147 2.29681294620007 -2.49730646214463 h

-6.52941416286428 2.22991847155006 0.22853500790013 h

0.00000000000000 2.18884024376465 -3.12288654687132 c

0.00000000000000 -4.22904725811963 -4.04536032578199 o

0.00000000000000 4.22904725811963 -4.04536032578199 o

**[DNTI]^+^**

$coord

0.00000000000000 0.00000000000000 5.99195696048093 i

0.00000000000000 0.00000000000000 -4.54296354236039 o

0.00000000000000 1.47188732219168 -0.30174977272553 c

2.51378220166008 -2.17412027569925 1.06118469716450 c

2.57313569316580 -4.08336297360798 1.81163892587750 h

4.79975509494393 -1.48082039794966 -0.65027828219451 c

4.63117252393844 -2.29577974927352 -2.52999399204245 h

6.53191118855401 -2.22998481143278 0.16639983880631 h

-2.51378220166008 2.17412027569925 1.06118469716450 c

-2.57313569316580 4.08336297360798 1.81163892587750 h

2.84383287089420 0.00000000000000 2.94534654769552 c

4.59757453490064 0.00000000000000 4.00949489861719 h

0.00000000000000 -1.47188732219168 -0.30174977272553 c

2.51378220166008 2.17412027569925 1.06118469716450 c

2.57313569316580 4.08336297360798 1.81163892587750 h

-4.79975509494393 -1.48082039794966 -0.65027828219451 c

-6.53191118855401 -2.22998481143278 0.16639983880631 h

-4.63117252393844 -2.29577974927352 -2.52999399204245 h

4.79975509494393 1.48082039794966 -0.65027828219451 c

6.53191118855401 2.22998481143278 0.16639983880631 h

4.63117252393844 2.29577974927352 -2.52999399204245 h

0.00000000000000 -2.18733277015050 -3.08614467352053 c

-2.84383287089420 0.00000000000000 2.94534654769552 c

-4.59757453490064 0.00000000000000 4.00949489861719 h

-2.51378220166008 -2.17412027569925 1.06118469716450 c

-2.57313569316580 -4.08336297360798 1.81163892587750 h

-4.79975509494393 1.48082039794966 -0.65027828219451 c

-4.63117252393844 2.29577974927352 -2.52999399204245 h

-6.53191118855401 2.22998481143278 0.16639983880631 h

0.00000000000000 2.18733277015050 -3.08614467352053 c

0.00000000000000 -4.22779642215375 -4.00934608434971 o

0.00000000000000 4.22779642215375 -4.00934608434971 o

**[(CH_3_)_2_F]^+^**

$coord

-7.21178698055431 -2.15036805233638 -0.08574713464112 c

-2.17569534244732 -2.33435253793977 0.06026251353845 c

-4.75985064336209 -3.29322082103004 0.95266954255819 f

-8.61079932230119 -3.26677823977091 0.90641391428433 h

-7.12078402132827 -2.49336484006848 -2.10205815235469 h

-7.13457023274653 -0.18340440452723 0.47623680718163 h

-2.15201241343958 -0.35879148015890 0.59651683566919 h

-2.16476119340744 -2.69932510314026 -1.95420588816326 h

-0.92137056735281 -3.52905450507286 1.14991156192670 h

**[(CH_3_)_2_Cl]^+^**

$coord

-2.74940855722662 0.12273921311639 -0.10373808284162 c

2.75257938294357 -0.07443095932565 0.06003815624264 c

-0.09698215038874 -1.53980428082857 1.40500068872604 cl

-4.39688948724873 -0.78324511501959 0.71978524410537 h

-2.58830312515236 -0.22025789915257 -2.11725951762158 h

-2.58708686276909 2.09393352910595 0.43060088596045 h

2.70062179364348 1.90412530812094 0.58916555387076 h

2.68619355139871 -0.40789985519255 -1.96038746537809 h

4.27927545479973 -1.09515994082437 0.97679453693595 h

**[(CH_3_)_2_Br]^+^**

$coord

-2.90592724284390 0.14581896324264 -0.12057657933605 c

2.91107894720162 -0.06631976017248 0.04837628090630 c

-0.10793487316150 -1.71288054257974 1.57056867818872 br

-4.58972636689088 -0.73085316537955 0.65692445602397 h

-2.69223038553339 -0.19477492064566 -2.12831171902648 h

-2.70913445250679 2.10811529291477 0.42972110142138 h

2.81350790147972 1.91078585170088 0.57145022650600 h

2.80157898293334 -0.41439056550041 -1.96635359489260 h

4.47878748932174 -1.04550115358045 0.93820115020878 h

**[(CH_3_)_2_I]^+^**

$coord

-3.08835274618120 0.16478022935070 -0.13716450911544 c

3.09499971831997 -0.06058910016267 0.04234325802871 c

-0.12120578424990 -1.92124517575004 1.76102927761576 i

-4.82088947470275 -0.65759238975392 0.59172156281031 h

-2.84883790426908 -0.17365133635600 -2.14244227541951 h

-2.86601404309598 2.12557695416154 0.40912449802697 h

2.97193184372001 1.91641738323474 0.56072465245956 h

2.96040057635575 -0.40406286408596 -1.97172641195501 h

4.71796781410314 -0.98963370063837 0.88638994754865 h

**[F_2_DNTF]^+^ (ground state)**

$coord

-0.41516332840997 0.00009459289085 4.63446378593041 f

-0.04389974677222 -0.00004864482538 -4.55658864718025 o

0.00785633339142 1.48825144576630 -0.32348581595427 c

2.49995132559086 -2.21374919650710 1.05546344467431 c

2.57283156272630 -4.07559034764483 1.90882288040558 h

4.85724766528326 -1.49100182021306 -0.60668880664510 c

4.68349613948191 -2.31190222442410 -2.48227496782468 h

6.57125747769032 -2.24093421029241 0.24251544913609 h

-2.46852349354219 2.18258514853313 1.09414360244774 c

-2.49694521617597 4.07130264526178 1.89832073879030 h

2.82022567800066 0.00025346516500 2.74757898661822 c

4.18465811439661 0.00033287445737 4.72660340958414 f

0.00798570391048 -1.48821773592402 -0.32339858479923 c

2.49979545385202 2.21406487096864 1.05531049982805 c

2.57273862273914 4.07601795761231 1.90841537986082 h

-4.79678918966581 -1.48230596886229 -0.53469645069279 c

-6.49959572704755 -2.21914219428434 0.34753636488055 h

-4.69954192207717 -2.29756972351397 -2.41843322263787 h

4.85711701242808 1.49123704646576 -0.60689556728375 c

6.57104824765233 2.24151308260617 0.24214113916846 h

4.68306708116726 2.31193475540554 -2.48253986038338 h

0.00413306326151 -2.19247902836923 -3.11286404915908 c

-2.58658931756518 -0.00001678972883 2.98144714469459 c

-4.54401569730814 -0.00012011893918 4.55486748295819 f

-2.46830145497073 -2.18270745091273 1.09427736219934 c

-2.49650779459687 -4.07137977219747 1.89858400692394 h

-4.79691452376916 1.48181222294718 -0.53482093804660 c

-4.69977085342855 2.29692943304786 -2.41862051855686 h

-6.49977519594662 2.21854342699537 0.34738631798686 h

0.00383972623145 2.19236643091508 -3.11300270770748 c

0.05790044160921 -4.23060284056691 -4.03180605859742 o

0.05718381186341 4.23052866816750 -4.03192348929910 o

**[F_2_DNTF]^+^ (*C*_2v_ transition state)**

$coord

-0.41516332840997 0.00009459289085 4.63446378593041 f

-0.04389974677222 -0.00004864482538 -4.55658864718025 o

0.00785633339142 1.48825144576630 -0.32348581595427 c

2.49995132559086 -2.21374919650710 1.05546344467431 c

2.57283156272630 -4.07559034764483 1.90882288040558 h

4.85724766528326 -1.49100182021306 -0.60668880664510 c

4.68349613948191 -2.31190222442410 -2.48227496782468 h

6.57125747769032 -2.24093421029241 0.24251544913609 h

-2.46852349354219 2.18258514853313 1.09414360244774 c

-2.49694521617597 4.07130264526178 1.89832073879030 h

2.82022567800066 0.00025346516500 2.74757898661822 c

4.18465811439661 0.00033287445737 4.72660340958414 f

0.00798570391048 -1.48821773592402 -0.32339858479923 c

2.49979545385202 2.21406487096864 1.05531049982805 c

2.57273862273914 4.07601795761231 1.90841537986082 h

-4.79678918966581 -1.48230596886229 -0.53469645069279 c

-6.49959572704755 -2.21914219428434 0.34753636488055 h

-4.69954192207717 -2.29756972351397 -2.41843322263787 h

4.85711701242808 1.49123704646576 -0.60689556728375 c

6.57104824765233 2.24151308260617 0.24214113916846 h

4.68306708116726 2.31193475540554 -2.48253986038338 h

0.00413306326151 -2.19247902836923 -3.11286404915908 c

-2.58658931756518 -0.00001678972883 2.98144714469459 c

-4.54401569730814 -0.00012011893918 4.55486748295819 f

-2.46830145497073 -2.18270745091273 1.09427736219934 c

-2.49650779459687 -4.07137977219747 1.89858400692394 h

-4.79691452376916 1.48181222294718 -0.53482093804660 c

-4.69977085342855 2.29692943304786 -2.41862051855686 h

-6.49977519594662 2.21854342699537 0.34738631798686 h

0.00383972623145 2.19236643091508 -3.11300270770748 c

0.05790044160921 -4.23060284056691 -4.03180605859742 o

0.05718381186341 4.23052866816750 -4.03192348929910 o

**[F_2_DNTCl] ^+^**

$coord

-0.00000153248771 0.00004325990948 5.36600627068124 cl

-0.00006851382897 -0.00008330606743 -4.52690009254079 o

0.00005016754658 1.48403884065569 -0.28996164994520 c

2.50225538553096 -2.19159532054451 1.07502951992788 c

2.56119662036951 -4.07784785073641 1.88013618502498 h

4.80918266948879 -1.48302697357035 -0.60819843655940 c

4.64886062204107 -2.30044612948728 -2.48619964073287 h

6.53223741606460 -2.22418439531771 0.22875147798314 h

-2.50220897989265 2.19162130907341 1.07495353381032 c

-2.56110413057419 4.07788781739481 1.88003082093730 h

2.75697196261833 -0.00004612657229 2.91957341093281 c

4.75916538342324 -0.00005737633339 4.43000007491787 f

-0.00000882248908 -1.48407294926883 -0.28992275785639 c

2.50220949386499 2.19160324241986 1.07514971279853 c

2.56099849354202 4.07780757326892 1.88037716457405 h

-4.80917414505784 -1.48323226564273 -0.60806648778394 c

-6.53220834357323 -2.22426120228229 0.22903960724125 h

-4.64892068945766 -2.30088678377598 -2.48596920932777 h

4.80931357144223 1.48323873179109 -0.60789239931549 c

6.53229089735191 2.22405473330642 0.22951498591410 h

4.64936116225446 2.30109262510979 -2.48573168727389 h

-0.00025495892529 -2.19074308756585 -3.08114087255633 c

-2.75695281597163 0.00011330308799 2.91955135943663 c

-4.75915904212916 0.00019076017209 4.42996164357900 f

-2.50219062973040 -2.19157833156982 1.07518367834284 c

-2.56103863011140 -4.07777021867435 1.88044059432150 h

-4.80922880568584 1.48303377371978 -0.60810618867941 c

-4.64917344737189 2.30065429856342 -2.48603760882811 h

-6.53224086378050 2.22397798160650 0.22911753181675 h

0.00010896242364 2.19063086566685 -3.08120904868312 c

-0.00061016575633 -4.23051841352609 -3.99874662032976 o

0.00034170886144 4.23036161518913 -3.99889656050784 o

**[(CH_3_)_2_DNTF]^+^**

$coord

0.68365192263414 27.16484972999767 23.80135195900626 f

8.10065523364110 26.85170432160530 17.79706560326634 o

4.47765954977840 26.60728829417867 15.56355457595093 o

0.41440747129021 26.42541733227945 14.28583740442742 o

5.86197705682938 26.80624108184850 17.79056058453135 c

4.05186391271919 26.94121803559202 20.01456546037870 c

0.19387773309444 24.38150800347974 19.98183245402316 c

-1.85629041654535 24.30336365457790 19.90686618643729 h

1.54919208980377 21.98673035287990 18.96498227602479 c

1.59277803156957 21.95816859868685 16.91057021206323 h

0.54372521431110 20.29214838234947 19.55158010929348 h

4.03644784822219 29.52778713749547 21.40283767311221 c

5.43350800276566 29.70364623837760 22.89726343543559 h

1.38278773850686 24.42961788446645 22.60157887008405 c

0.52402815160613 22.73978982896075 24.68607479520960 c

1.36767024470444 26.79270476976773 18.78815358815310 c

4.13582489961450 24.59968741274765 21.78179655932160 c

5.53329930283529 24.71253193167409 23.28206466350438 h

1.35413897171810 31.57324042760937 18.22816281136518 c

0.28042317449159 33.29546996970520 18.55522680516337 h

1.40136306635666 31.29074168872592 16.19326022342634 h

4.24005166583480 22.13703335670699 20.19242687460197 c

4.61019740966962 20.51768327203979 21.40302600302863 h

5.76167775693238 22.19422564564742 18.81187077118154 h

1.87936194026331 26.58596806238441 15.97137415702999 c

1.27599505848047 29.71037412043175 22.19495095470248 c

0.34305292291182 31.66337392208982 23.99884921086595 c

0.09410750743105 29.30870996506697 19.60258607659825 c

-1.95731739024943 29.29112684038397 19.52302831366717 h

4.04680480528931 31.71939051871743 19.45334167054286 c

5.57157966116480 31.50968190720486 18.09205222755626 h

4.35004061008450 33.51882961506868 20.39972726383341 h

-1.68035253595379 31.47633788812311 24.32002726113435 h

0.68875054289663 33.53095862007491 23.19904587180696 h

1.34621707241228 31.55433335342601 25.79173128862086 h

0.87895764522927 20.78221748826856 24.14845679241266 h

-1.49492410943327 22.94476495168022 25.02492347589885 h

1.56319973730631 23.11374139671840 26.42196143717204 h

**[(CH_3_)_2_DNTCl]^+^**

$coord

0.32765877466908 27.21648243621750 24.57181774547504 cl

8.03607593899264 26.86070930126256 17.93409710008409 o

4.41722750243517 26.61613058746680 15.69738880998571 o

0.35452496584610 26.43594141247262 14.42387860249930 o

5.79687701490289 26.81506855861500 17.92623274059025 c

3.98442499371559 26.94999690873953 20.15464595073909 c

0.15800393584120 24.34347262048667 20.06578190787264 c

-1.89046648071298 24.23325467975762 19.97211610152452 h

1.55740905504661 22.01796708273415 18.94137063221910 c

1.60059390980327 22.07257155477519 16.88808200134761 h

0.58247553403362 20.28173116134062 19.45047313674422 h

3.99834840553459 29.57667423458721 21.48146378260879 c

5.40872788666749 29.78134468089348 22.96020439432885 h

1.33790400259561 24.25756769862513 22.71761781156890 c

0.62267693182084 22.16873147394847 24.53308150688361 c

1.30631701192267 26.80313303030088 18.93062285724609 c

4.09797925976548 24.55955173551868 21.86559301588209 c

5.51100032840305 24.63959214887258 23.35424393126299 h

1.36910216708152 31.53963359232003 18.20951304011140 c

0.32532385791905 33.29333554904631 18.44938650602370 h

1.42284859878985 31.17247744547499 16.18905917506535 h

4.24690252264318 22.16682186451120 20.16922671197340 c

4.65625897355803 20.50592139691325 21.30764271494088 h

5.76108358193766 22.30627139143271 18.78767984349401 h

1.82053887870553 26.59586232002231 16.10928084324446 c

1.22576507410825 29.89633842412049 22.28407647100824 c

0.42235724276746 32.20750008572733 23.76064209959990 c

0.05876147440388 29.36023324504349 19.67968167585731 c

-1.99210262755618 29.37314348506450 19.57573795315510 h

4.05866267069592 31.68719248077693 19.43922587067539 c

5.58142631144259 31.39915380957814 18.09134838152626 h

4.39716649272867 33.51670941710379 20.31150010630015 h

-1.59984131657717 32.18017534445175 24.14576882355516 h

0.82710243015045 33.90325815576945 22.67222119316548 h

1.44648284429510 32.34779727968161 25.54092529778948 h

1.10244786233593 20.34365108065575 23.71864334781442 h

-1.40087374208879 22.17146968684307 24.91227848417083 h

1.64321723159357 22.34573863988724 26.31201533249874 h

**[(CF_3_)_2_DNTF]^+^**

$coord

0.71189366084464 27.16171990212476 23.73403903611093 f

8.12275386879251 26.84804728737697 17.76298061889423 o

4.49979239065252 26.60505980899153 15.52355321635580 o

0.43436012184596 26.42385720618432 14.24319322690462 o

5.88709040147200 26.80273367135268 17.74601361693943 c

4.07322857206579 26.93706461850785 19.97357225626757 c

0.20195903639282 24.37158919867194 19.92959290256885 c

-1.84752565191840 24.29672949076661 19.86709139681686 h

1.56915341297802 21.98187489129511 18.90797864212712 c

1.60352940659340 21.97861316979468 16.85318294153035 h

0.57293743072336 20.28275491741264 19.48709452711594 h

4.06785851181198 29.53008861498667 21.36579253717182 c

5.45181507724391 29.70244126548199 22.87109521753973 h

1.40738606159015 24.48414428493168 22.53246533887503 c

0.48198456860137 22.84438613975238 24.76450457472696 c

1.38598586693309 26.79078556149854 18.74351969435676 c

4.16440586081678 24.58591191546692 21.73935854433256 c

5.55145943880599 24.69527467980811 23.24783607547091 h

1.39064506560140 31.57144783198368 18.17332783284054 c

0.32784961743962 33.29980863082525 18.48359858993387 h

1.43879787210177 31.26005917304955 16.14268693133743 h

4.25832782381858 22.12889869630974 20.13439899981184 c

4.62574639181201 20.50231534653281 21.33117851573829 h

5.78363486685761 22.21096373604539 18.75927213806345 h

1.90244580179595 26.58352906360931 15.92216411130697 c

1.30511520192217 29.64482832730938 22.13865103107229 c

0.30364129578872 31.57162627553434 24.09199357342020 c

0.10817121455391 29.31593634398065 19.54913615127204 c

-1.94241297383031 29.30361582876573 19.47753206981951 h

4.07635008192590 31.71814452351190 19.40839022041949 c

5.61174628267911 31.49003175899401 18.06216395642257 h

4.37138407755084 33.51970661303118 20.34733108428006 h

-2.13137702159546 31.14338941726648 24.56137887365218 f

0.56174793767108 33.90613063812999 23.17591217026897 f

1.60513511644769 31.38858280802706 26.23816442786979 f

0.86372129851284 20.40987030924179 24.23210170036151 f

-1.97512881449047 23.21933038783872 25.15771636845705 f

1.75478032740854 23.43135146116953 26.85454609859537 f

**[(CF_3_)_2_DNTCl]^+^**

$coord

0.34713212916643 27.21432075834682 24.53343295605000 cl

8.05240714414426 26.86215497868246 17.90441546201235 o

4.43267946119367 26.61354827564698 15.66496950886053 o

0.36698700249675 26.43100247164752 14.39317169943429 o

5.81591304427592 26.81348697476916 17.88871338505291 c

4.00089133500882 26.94781261293062 20.12254365269172 c

0.17030569964225 24.32972573528593 20.01904831729296 c

-1.87700226604731 24.21654408801807 19.93321708679742 h

1.58936543540413 22.02295560097943 18.87418052950684 c

1.62061872849704 22.11510011087088 16.82226921559351 h

0.63431259201943 20.27415202898324 19.36233540065168 h

4.02787193104874 29.58484594961891 21.43985230795377 c

5.43214891805292 29.79415522542812 22.92189186487138 h

1.35823063974686 24.30792218966903 22.66920397553189 c

0.56860200214570 22.23705638079702 24.61335862576290 c

1.32100333376585 26.80050732193795 18.89784755456798 c

4.12847483865614 24.54548296785633 21.82654858832038 c

5.53662756223107 24.62167105779996 23.31836025319821 h

1.40260889646555 31.52293771673243 18.13955211961715 c

0.37698639670529 33.28669691346125 18.34722489483716 h

1.45170137939176 31.11099036928314 16.12798435777012 h

4.27869045195915 22.17387896668466 20.09600074645364 c

4.70250551759558 20.49819705835043 21.19976417678901 h

5.78944658125887 22.36047214930985 18.71697375709221 h

1.83741564743259 26.59198065825254 16.07114851871254 c

1.24832660060853 29.84011841774341 22.24275191421562 c

0.39299406663663 32.15153909166984 23.85956966028365 c

0.06995658376706 29.36778288247601 19.63038120393830 c

-1.97995002667839 29.38471937261606 19.53335013303532 h

4.08906581319271 31.67236217042409 19.36994441262091 c

5.61487257247283 31.34588532458050 18.03486730457220 h

4.43343139104906 33.51128049165541 20.21054977866990 h

-2.07460978215830 31.96385324105824 24.38478938342417 f

0.78023377631493 34.33309346718511 22.67993025875608 f

1.67938138385398 32.18891376203738 26.03681452582835 f

1.12578912894214 19.92304643306782 23.81683216495214 f

-1.92510781948934 22.35837402790880 25.01924648991212 f

1.76008140944803 22.62407655179639 26.81147302341749 f
