# Supplementary material for: Structural proof of a [C–F–C]+ fluoronium cation
Source: Nat Commun. 2021 Sep 6;12:5275. doi: 10.1038/s41467-021-25592-6 (PMC8421340; doi:10.1038/s41467-021-25592-6)
Supplement: Supplementary file 4 — Article File - Editor’s Summary [file 41467_2021_25592_MOESM4_ESM.pdf]

Description of Additional Supplementary Files

File name: Supplementary Data 1
Description: Cartesian coordinates of quantum-chemical optimized structures

File name: Supplementary Movie 1
Description: Animations of selected modes of the double-norbornyl type fluoronium ion
